# Supplementary figures and images for: Manipulating multi-level selection in a fungal entomopathogen reveals social conflicts and a method for improving biocontrol traits
Source: PLoS Pathog. 2024 Mar 25;20(3):e1011775. doi: 10.1371/journal.ppat.1011775 (PMC10994555; doi:10.1371/journal.ppat.1011775)

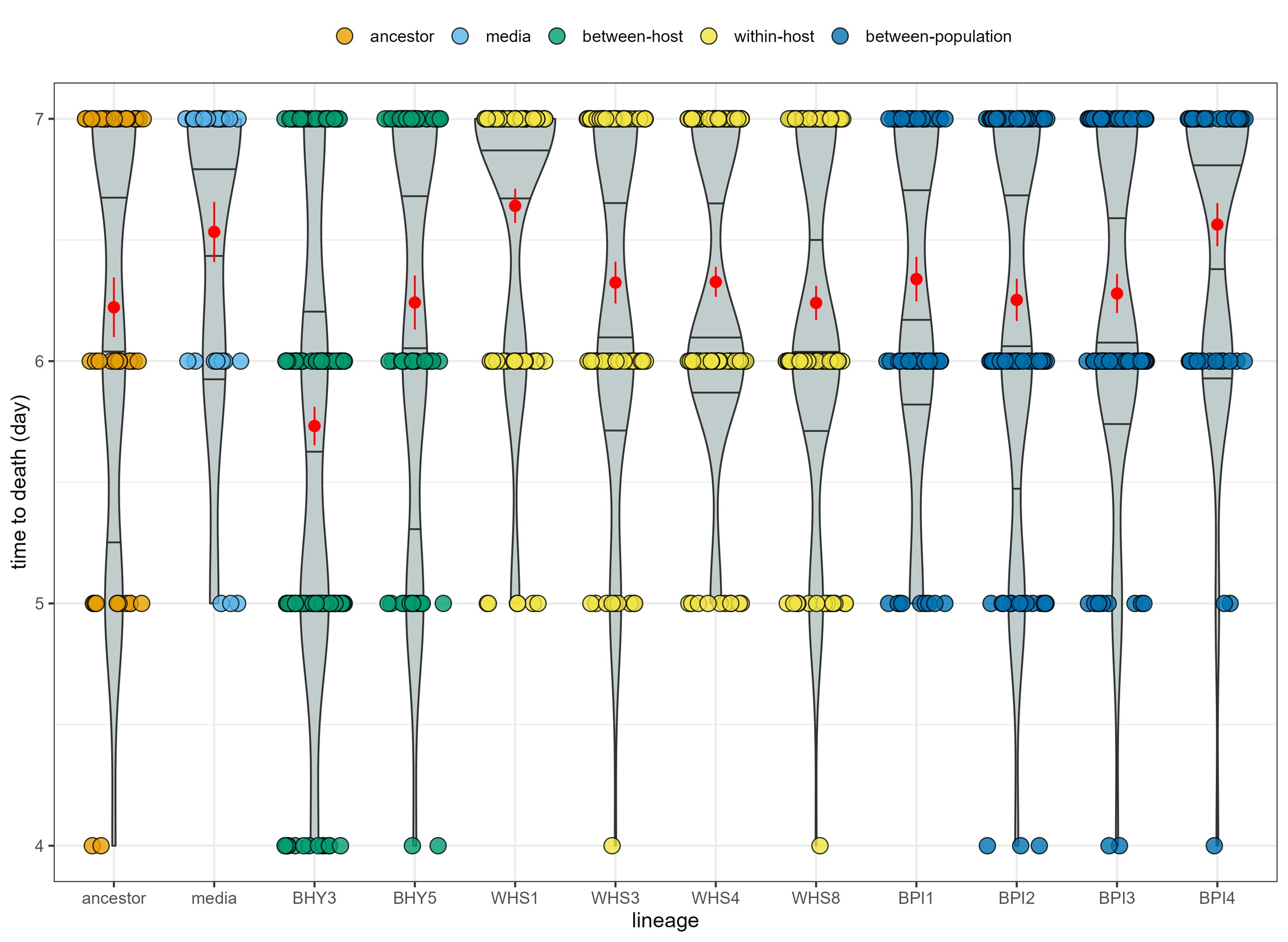

Supplement: S1 Fig — Red dots represent the mean with standard errors. (TIF) [file ppat.1011775.s001.tif]

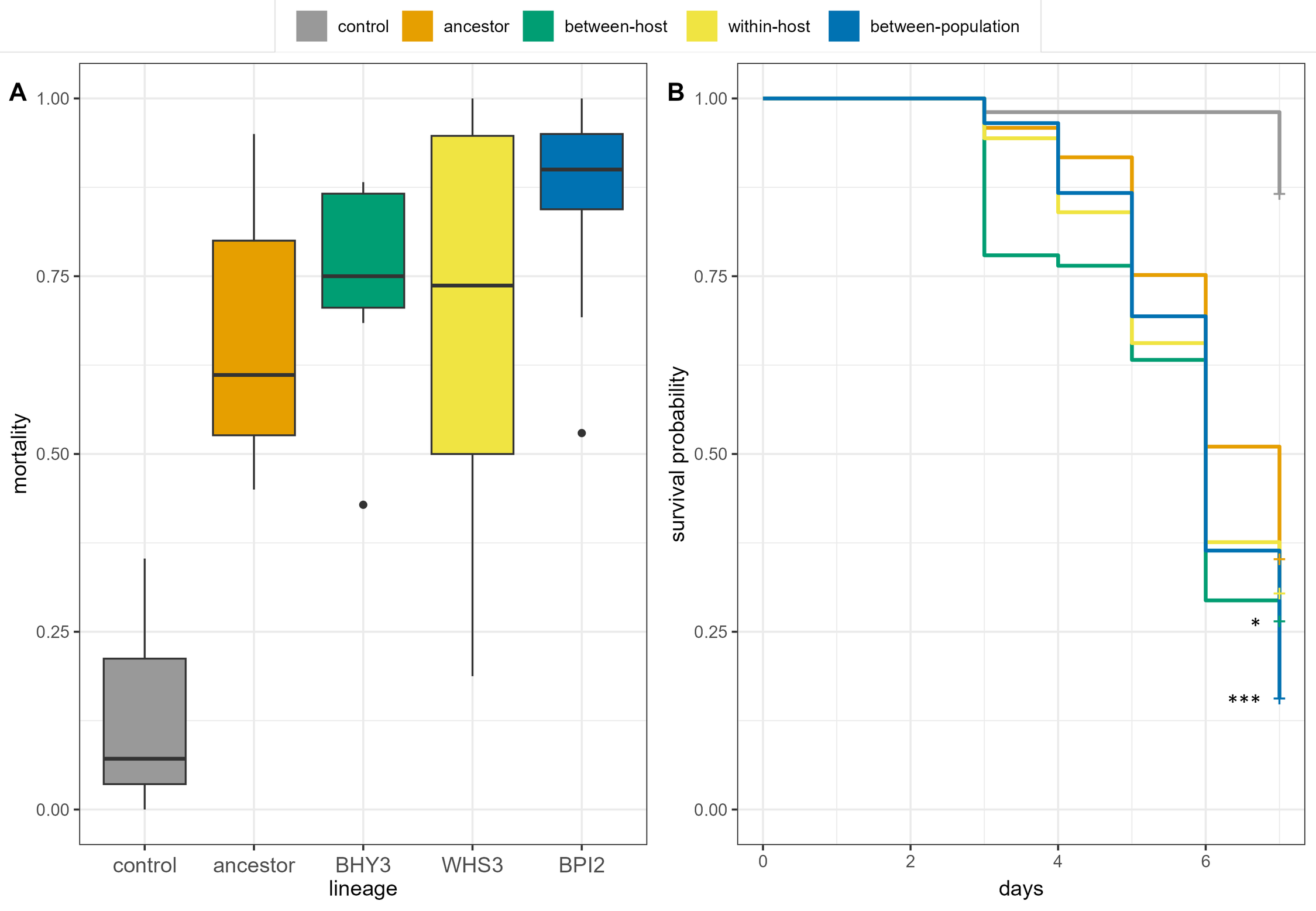

Supplement: S2 Fig — Total mortality of B. brassicae after 7 days of exposure to selected lines of A. muscarius (A). Boxplots showing median, first and third quartiles, whiskers are 1.5 * interquartile range (IQR). Data beyond the end of the whiskers are outliers. Mean survivorship curves of B. brassicae over 7 days after treatment with selected lines of A. Muscarius (B). Significance levels for survival model: ***: p < = 0.001; *: p < = 0.05. (TIF) [file ppat.1011775.s002.tif]
