# Supplementary material for: Manipulating multi-level selection in a fungal entomopathogen reveals social conflicts and a method for improving biocontrol traits
Source: PLoS Pathog. 2024 Mar 25;20(3):e1011775. doi: 10.1371/journal.ppat.1011775 (PMC10994555; doi:10.1371/journal.ppat.1011775)
Supplement: Attachment — Submitted filename: response_to_reviewers_PLOSpathogens_0602.docx [file ppat.1011775.s003.docx]

Centre for Ecology and Conservation

University of Exeter

Penryn, Cornwall, TR10 9FE

[z.erdos@exeter.ac.uk](mailto:z.erdos@exeter.ac.uk)

Zoltan Erdos PhD

Dear Prof Alex Andrianopoulos and Prof Michael Malim,

Thank you for the opportunity to revise our paper “*Manipulating multi-level selection in a fungal entomopathogen reveals social conflicts and a method for improving biocontrol traits”* [PPATHOGENS-D-23-01852]. We thank the reviewers for their insightful comments and suggestions.

The response to each individual comment is included below directly followed by a proposal of addressing each concern. Response to reviewer comments are in blue, with all changes in the manuscript preceded by line number (revised manuscript, track changes on). The revisions have been approved by all authors. The changes are marked in the manuscript with track changes mode in MS Word.

Yours sincerely,

Zoltan Erdos

**Reviewer #1:** This is an interesting research article on multi-level selection in a fungal entomopathogen to understand the trade-offs in life history traits while trying to increase virulence. Overall, the research has merit but there are a few concerns that must be addressed. **1)** The authors claim that virulence of fungal parasites typically fails to increase when passed through their insect host. However, this claim is contrary to what the literature suggests. Research has shown that passage of an entomopathogenic fungus through its insect host increases fungal virulence. Please acknowledge this information and then provide the gap in literature you are trying to fill (Example paper: Evison, et al. 2020. J. Evol. Bio. 28:179-188).

*Evolution of virulence in fungal entomopathogens.*

*There should be a distinction made between one passage to restore virulence of a potentially attenuated strain and serial passage to increase virulence via selection of allelic variation. Rapid recovery of traits may be related to DNA methylation (eg Hu and Bidochka 2020). In terms of artificial selection the efficacy of passage on selecting for virulent fungal variant is mixed at best. We have considered this topic in detail previously in a full-length review on passage of insect pathogens (Raymond and Erdos 2022). In brief, quite a few studies that aimed to increase fungal virulence via passage failed to do so (Scully & Bidochka, 2005; Scully & Bidochka, 2006; Valero-Jimenez et al., 2017). There are some successful examples of serial passage increasing virulence (Adames et al., 2011; Evison et al., 2015). In one of these case (Adames et al 2011) there was not direct selection for virulence so this was almost accidental. In summary – it has been possible to increase EPF virulence previously but success is often poor and selection designs are not informed by theory- which could explain why they have had haphazard results.*

*We could not find the specific Evison et al 2020 study referee 1 discussed here- perhaps this was confused with the Evison et al 2015 study?* *We had cited the latter.*

*We have added a paragraph at line 92:*

*Passage of invertebrate pathogens is a commonly used method to restore or maintain virulence. However, distinction should be made between a single passage to restore virulence of a potentially attenuated strain and serial passage to increase virulence via selection on allelic variation. Rapid recovery of traits may be related to DNA methylation (Hu & Bidochka, 2020). In terms of artificial selection, the efficacy of passage on selecting for virulent fungal variants is mixed at best (Raymond & Erdos, 2022). Several studies that aimed to increase fungal virulence via passage have failed to do so (Scully & Bidochka, 2005; Scully & Bidochka, 2006; Valero-Jimenez et al., 2017). There are some successful examples of serial passage increasing fungal virulence (Adames et al., 2011; Evison et al., 2015), in one of these cases increased virulence was a result of simple passage through the host (Adames et al., 2011). These results show that it is possible to increase the virulence of entomopathogenic fungi (EPF) via artificial selection, but success is often poor and selection designs have not informed by theory, which could explain the conflicting results seen to date.*

**2)** Typically, experimental evolution studies do not involve several rounds of UV mutagenesis. Since UV mutagenesis was performed, the results should be interpreted with caution. For example, in lines 477-481, the genomic flexibility of the commercial strain of Akanthomyces muscarius is highlighted, but the observed genomic variation could be a result of UV mutagenesis.

*Mutagenesis and genetic variation in experimental evolution.*

*There is no standard practice here for experimental evolution studies. A key conceptual point is that mutation supply (vital for evolutionary change) is dependent on mutation rate and population size. Experimental evolution of bacteria in culture- with large population sizes – does not typically require additional mutagenesis. Nevertheless, Lenski’s classic E. coli experiments show us that mutator strains with high mutation rate do very well in these short evolutionary time scales.*

*In contrast, pathogen passage (with fungi or bacteria) involves small population bottlenecks at infection- this can purge genetic diversity (Scully & Bidochka, 2006). We have shown previously that high mutation rate is very important in generating selectable variation for bacterial passage in insects (Dimitriu et al 2023).*

*In applied “Directed Evolution” of insect pathogens mutagenesis via UV or mutagens such as EMS is extremely common. Zhao et al. (2016) used UV mutagenesis and screened >6000 colonies to identify a virulent mutant.*

*We have discussed the source of the genetic variation in this study. In the process of characterising the evolved and ancestral lines, we resequenced the ancestor and this showed us significant standing variation in the ancestral line (see figure 5). Whilst we hoped to increase genetic variation by UV mutagenesis, we cannot quantify precisely how much this increased genetic variation, partly due to the unexpectedly high standing variation. In any case, we would argue that the use of UV mutagenesis does not invalidate our findings.*

**3)** Considering that Akanthomyces muscarius is a filamentous fungus, what is the biological interpretation of the social evolution theory? In filamentous fungi, cells are not discrete entities but are interconnected. So, the occurrence of cheater cells needs a more biologically relevant explanation. For example, heterokaryosis is commonly known to occur in filamentous fungi in which two or more genetically distinct nuclei are present in the same cell (Gambhir, et al. 2022. Msphere, 7:e00087-22). Please see additional comments below.

*Social evolution theory has been applied successfully to aggregations of microbes in biofilms, for example. Spatially separated clones in aggregations can make cheating less favourable- this is a well-explored topic. (E.g. Zhou et al. (2014);Granato et al. (2018)). However, spatial structure of microbes has to be relevant to the diffusion rates of secreted public goods, or for timing of sporulation, for nutrients. In other words, if nutrients can diffuse effectively between patches of clones, then cheating can still occur. In brief, there is nothing particular about filamentous growth that makes social evolution theory inappropriate for fungi.*

*We are aware that heterokaryotic forms of EPF have been described but do seem to be rare- this could act to decrease relatedness and enhance success of cheaters. Nevertheless, social evolution theory was originally formulated to explain cooperative behaviour in diploid animals so this is not a basis for rejecting our use of this theory.*

**Reviewer #2**: In their manuscript entitled Manipulating multi-level selection in a fungal entomopathogen reveals social conflicts and a method for improving biocontrol traits, Erdos et al. use serial-passaging to try and reveal trade-offs in key pathogen traits important for infectivity and virulence. Interestingly the authors do not discover any of the normally predicted trade-offs in obligate-killing pathogens, but describe a phenotypic change to earlier sporulation in a between group selection treatment. The findings are novel and exciting by bringing and using social evolution theory to entomopathogenic fungi, and as such should be of interest to a wide audience.

The manuscript is well written and figures easy to follow. Apart from some minor comments listed below, my main concern with this manuscript is that the use of developed terminology is confusing, and makes the introduction and the links to the experimental plan difficult to follow. For example, when referring to between group treatment in the abstract, is that then the between populations or between host treatment, I understood the former but it was not perfectly clear. Furthermore, the three different treatments are referred to as speed of kill treatment, infectivity selection, and yield treatment, but the link to the different scales of selection described in the introduction as: between populations, between hosts, and within hosts are not easy to follow. The different names of the treatments are introduced at the end of the introduction, but should be more clearly defined with links to the scale of selection (i.e. between, within hosts or between populations). When these terms are used in the first paragraph of the results section on page 10, it is not immediately clear what the different treatments involve. As a reader, I found figure 7 very informative, and I suggest to re-organize the manuscript to introduce figure 7 much earlier, maybe as a new first figure and specifying the links to the scales of selection, so the experimental set-up is more easily linked to introduction. For example, on L192-193 it is stated that: “Total mortality is sometimes described as infectivity, but we will avoid this term to prevent confusion with the infectivity selection regime.”. But at this point in the text, the infectivity selection regime is not clearly defined. I assume this is defined on L149-151, but to make it more clear for readers when defining these experiments this sentence could start with: “Infectivity selection regime: We predicted … etc.” or similar to make it unambiguous for readers.

*We simplified the terminology used in the manuscript by changing treatment names from infectivity, speed and yield treatments to between-population, within-host and between-host treatment respectively. The changes have been made to all occurrences of treatment names (graphs, table).*

*Line29: changed to accommodate new treatment names and easier following: “stock we selected for speed of kill, parasite yield or infectivity by manipulating competition within and between hosts and between-populations of hosts over 7 rounds of infection.”*

*Line39: between-group changed to between-population to avoid confusion.*

*Figure 7 from the methods section was moved to the introduction as suggested*.

*The individual lineage names belonging to each treatment (e.g BPI1-BPI4) are now also mentioned in the new figure 1 caption, to introduce them earlier. The lineage names belonging to the within-host treatment have been renamed from BHS1, BHS3, BHS4, BHS8 to WHS1, WHS3, WHS4, WHS8 respectively.*

If the methods were presented before the results section, the manuscript would be much easier to follow, but since this is not the structure of this journal, care must be taken to make it comprehensible for readers to go straight from the introduction to results. There are numerous examples of details referred to in the results that have not been properly introduced, for example on line 205 where specific lineages are mentions, BPI2 etc., but what does BPI stand for? This is only mentioned in the figure legend. One way to remedy this is to add a new first paragraph (maybe including a reference to a new figure 1 as mentioned above) at the start of the results, providing more detail of the experimental set-up. Alternatively, the final paragraphs of the introduction could be reworked to make the experimental set-up more easily comprehensible.

*We’ve added a line to the beginning of the results section to help orientate the reader and reiterate predictions for treatments.*

*Line 209: Added “We hypothesized that the between-population (BP) treatment would result in strongest selection on infectivity, that the between-host (BHY) treatment would most efficiently select for increased pathogen yield and the within-host treatment (WHS) would either increase speed of kill or lead to cheating and loss of virulence.”*

If I understand the experimental protocol correctly, the infected and chosen cadavers were collected, surface sterilized, and placed on SDA agar. After fungal growth and sporulation on the agar, conidia were harvested and used to inoculate the next round. This imply that the fungi effectively alternates between growing on and inside an aphid and then on agar in each round. I perfectly sympathize with the authors for this methodology, but would like to see a discussion of this fact incorporated in the manuscript, as this may have an effect – especially on the strength of selection imposed by the Aphid environment.

*This is correct, fungi effectively alternates between in vivo and in vitro growth in each round. The in vitro propagation was unavoidable to reach high enough propagules for the next round of infection. Previous studies have shown that this does not prevent meaningful adaptation to hosts (Dimitriu et al., 2023; Matthews et al., 2019). Furthermore, the media control line was set up to control for any confounding effects.*

*Added lines at 512: “The need to propagate fungi in vitro prior to infection means that there might be alternating selection acting against adaptation to the in vivo environment. Previous studies have shown that this does not prevent meaningful adaptation to hosts (Dimitriu et al., 2023; Matthews et al., 2019). The media selection treatment was set up to control for any confounding effects. Furthermore, the ability to grow and sporulate well in vitro is also key for successful development of EPF as biocontrol agents, therefore the complete absence of this growth environment might be negative for this purpose.”*

**Part III – Minor Issues: Editorial and Data Presentation Modifications**
Please use this section for editorial suggestions as well as relatively minor modifications of existing data that would enhance clarity.

**Reviewer #1**:

- Line 26. As explained in point 1), this is contrary to what the literature suggests.

*Please see response on evolution of virulence in fungal entomopathogens above.*

- Line 29. This sentence needs to be modified to ensure accuracy. The experiment was not started with a genetically diverse stock, but with the ancestor A. muscarius. To increase genetic variation, random mutagenesis was induced later.

*Please see response on genetic variation above.*

- Lines 90-98. Since this paper is focused on fungal enothomopathogens, virulence should be discussed in this context instead of solely relying on entomopathogenic nematodes. There is ample of evidence that suggests that passage of an entomopathogenic fungus through its insect host increases fungal virulence. Please acknowledge this information and then provide the gap in literature you are trying to fill.

*We have referenced a wide range of sources on different insect parasites and pathogen (see our response on evolution of virulence in fungal entomopathogens above). We have also referred to a number of passage experiments on entomopathogenic fungi as well as our recent review on virulence and social evolution theory in insect pathogens. This MS is however the first, to our knowledge, that has explicitly tested for social conflicts in a fungal pathogen. This means that we do have to rely on studies of other pathogens for examples.*

- Lines 100-115. Although this is a good explanation of the social evolution theory. Considering that Akanthomyces muscarius is a filamentous fungus, what is the biological interpretation of the social evolution theory? Please refer to point 3) above for more details.

*Addressed in major comments above.*

- Line 253. Uppercase 'I' should be replaced with lowercase in 'I.e'

*Corrected to i.e*

- Line 513. What is the required conidial concentration?

*This section of the manuscript refers to how we prepare spore suspension in general. The required concentrations changed depending on the use of the suspension. The relevant conidial concentration used were specified in the section where the specific assay is detailed. For example, virulence assays were carried out with spore suspensions of 3x106 conidia/ml (line 664), spore production assays were inoculated with 1x107 conidia/ml (line 688).*

- Lines 530-531. Please mention the within-host selection.

*Change the corresponding line (605) to “between-population selection for infectivity, within - host selection for speed of kill and between-host selection for yield”*

- Figure 3 B. The yellow plus signs are not visible. Please consider increasing the thickness of the symbol and/or changing the color.

*We have changed the symbol of the points to a filled rhombus for better visibility.*

- Lines 477-481. The observed genomic variation could be a result of UV mutagenesis.

*Please see response on genetic variation above.*

**Reviewer #2: Minor comments:**
L54 – dependent on how?

*Modified line 55 to read:” … significant differences in timing and production of spores, dependent* ***on*** *how we manipulated scales of selection…”*

L164 – While several?

*Modified line 194 to read: “While several lineages…”*

L201 – Please consider to provide the phenotypic effect sizes.

*We’ve added some additional information on phenotypic effect sizes regarding total mortality as below:*

*Line 240 was modified as: Changes in overall virulence were predominantly related to total mortality rather than the timing of death although phenotypic effect sizes were modest: the largest changes were in lineages in the between-host treatment with 2-fold increase in total mortality relative to the ancestor.*

*We refer to Figure 2B and C for more information of effect sizes on total mortality. Furthermore, effect sizes from the survivorship analysis are highlighted within the text as hazard ratios in brackets.*

*We have added 95% confidence intervals after hazard ratios in brackets “(1.84, 95% CI [1.38, 2.45])”*

L247 – Could significantly higher early sporulation be replaced with significantly earlier sporulation?

*Although we believe that this would be correct, we have not measured spore production any earlier than 3 days, therefore we cannot say that e.g day 1 sporulation supports this case. Therefore, we propose to keep the current language.*

L277 - Figure 3 – Is it possible to add which linear model estimates are significantly different from the ancestor as indicated on L269?

*Added significance levels to Figure 3A*

L319 – How many SNPs were inside or in close proximity to coding regions? How were the candidate SNPs presented in Table 1 chosen?

*The SNPs and INDELs (and region up and downstream from the mutation) that were considered ‘Convergent’ as described on line 319, were manually blasted against NCBI protein database. Table 1 collates the information for convergent mutations which have meaningful annotation ie have hits above 80% sequence similarity. We only checked if convergent mutations were in ORFs for the mutations in Table 1.*

*Modified table 1 caption to clarify this: “Regions containing convergent variants and their best respective blast hits. All mutations were located within coding regions in putative ORFs.”*

L359-360 – I did not read the introduction as this being your primary hypothesis, please consider adjusting in line with my main comment above regarding the use of developed terminology and presentation of hypotheses and experimental design.

*We’ve included a line re-introducing our hypotheses at the beginning of the results section, as described above. We’ve modifed the line in question as follows:

Line404: “Our hypothesis was…”*

L379 – cells that are sporulating?

*We have clarified this and changed to:*

*Line 424: “it is a switch away from growth that can free up resources for actively dividing cells that have not yet committed to sporulation.”*

L385-387 – How does this reduced competition play out if the early sporulating variant has a head-start and has grown to a bigger biomass when the late sporulation variant enters the niche? Does this change the trajectory of the degree of the tragedy of the commons?

*We have not tested this, but I would expect strong effects of competitive exclusion by the first colonizer. Even more so in a spatially structured case, such as an insect?*

*Also, available resources at the point of inoculation would be reduced due to already germinating spores, which might confound any competitive effects? These assays used a spot inocula on the centre of an agar plate and growth stops well before reaching the sides of agar plates due to switch to sporulation…*

L418 – Which regulatory genes?

*Edited to “These changes were accompanied by mutations in a number of putative regulatory genes (i.e. transcription factors and zinc finger proteins)”*

L507 – How many transfers of the fungal isolate on media was performed prior to the experiments?

*Two transfers. The isolated ancestor was stored on porous beads in cryovials at -80 °C. One bead was removed in sterile conditions to inoculate an SDA agar slant that after 10 days of growth at 23 °C was kept in 4 °C for roughly 2 months. Spore suspensions for experiments were obtained by using a sterile loop to inoculate SDA agar plates from this agar slant. This two-step protocol follows that described in D. Chandler 1994 and aims to minimize subsequent culturing in vitro.*

*Modified text from Line 555:
“The ancestral strain of A. muscarius was isolated from Mycotal (Koppert, NL) and stored on porous beads in cryovials at -80 °C. One bead was removed in sterile conditions to inoculate an SDA agar slant that after 10 days of growth at 23 °C was kept in 4 °C for roughly 2 months. Spore suspensions for selection and specific assays were obtained by using a sterile loop to inoculate SDA agar plates from this agar slant. SDA plates were incubated in darkness at 23±1 °C for 10-14 days. This two-step culturing protocol aims to minimize the alteration of fungal phenotypes by subsequent culturing in vitro (Chandler, 1994). Evolved lineages were stored and cultured as described above. Conidial suspensions were prepared by agitating mycelia in SDA plates with 'L-shaped' spreaders after the addition of 10 ml of 0.01% v/v Triton X-100. The suspensions were then passed through sterile cheesecloth to remove any hyphal fragments. Suspensions were enumerated using Fastread102 counting chambers (Kova International, USA) and adjusted to the required concentration in sterile water with 0.01% Triton X-100. This method was used for all enumeration of conidia.”*

*Added reference (84): Chandler D. Cryopreservation of fungal spores using porous beads. Mycol Res. 1994;98(5):525-6.*

L578 – after the experiments, were the fungal isolates isolated and preserved for long-term storage in a freezer or maintained on agar, and if so for how many transfers before being assessed for changes in pathogen life history traits? Please add a bit more information on how the ancestor and selected lines were maintained (or immediately used) to assess phenotypic changes, as just a few in-vitro transfers on agar can alter the phenotype of fungi.

*The above described two step culturing method was used for both the ancestor and evolved lineages. Evolved lineages were reisolated (aphid host and agar plate for the in vivo and media treatments, respectively. The isolated lineages were stored on porous beads at -80 °C. Fungal cultures for assays were prepared as described in the above two step process. One bead removed from freezer stocks used to inoculate an agar slant that was kept at 4 °C for roughly 2 months. SDA agar plates inoculated from the agar slant to inoculate SDA plates for assays.*

Adames, M., Fernández-Ruvalcaba, M., Peña-Chora, G., & Hernández-Velázquez, V. M. (2011). Effects of passages through a suitable host of the fungus, Metarhizium anisopliae, on the virulence of acaricide- susceptible and resistant strains of the tick, Rhipicephalus microplus. *Journal of Insect Science*, *11*(1), 21. <https://doi.org/10.1673/031.011.0121>

Chandler, D. (1994). Cryopreservation of fungal spores using porous beads. *Mycological Research*, *98*(5), 525-526. <https://doi.org/https://doi.org/10.1016/S0953-7562(09)80472-3>

Dimitriu, T., Souissi, W., Morwool, P., Darby, A., Crickmore, N., & Raymond, B. (2023). Selecting for infectivity across metapopulations can increase virulence in the social microbe Bacillus thuringiensis. *Evolutionary Applications*, *16*(3), 705-720. <https://doi.org/https://doi.org/10.1111/eva.13529>

Evison, S. E. F., Foley, K., Jensen, A. B., & Hughes, W. O. H. (2015). Genetic diversity, virulence and fitness evolution in an obligate fungal parasite of bees. *Journal of Evolutionary Biology*, *28*(1), 179-188. <https://doi.org/https://doi.org/10.1111/jeb.12555>

Granato, E. T., Ziegenhain, C., Marvig, R. L., & Kümmerli, R. (2018). Low spatial structure and selection against secreted virulence factors attenuates pathogenicity in Pseudomonas aeruginosa. *ISME J*, *12*(12), 2907-2918. <https://doi.org/10.1038/s41396-018-0231-9>

Hu, S., & Bidochka, M. J. (2020). DNA methyltransferase implicated in the recovery of conidiation, through successive plant passages, in phenotypically degenerated *Metarhizium* [10.1007/s00253-020-10628-6]. *Applied Microbiology and Biotechnology*, *104*(12), 1-13. <https://doi.org/10.1007/s00253-020-10628-6>

Matthews, A., Mikonranta, L., & Raymond, B. (2019). Shifts along the parasite-mutualist continuum are opposed by fundamental trade-offs. *Proceedings of the Royal Society B-Biological Sciences*, *286*, 20190236. <https://doi.org/10.1098/rspb.2019.0236>

Raymond, B., & Erdos, Z. (2022). Passage and the evolution of virulence in invertebrate pathogens: Fundamental and applied perspectives. *Journal of Invertebrate Pathology*, *187*, 107692. <https://doi.org/https://doi.org/10.1016/j.jip.2021.107692>

Scully, L. R., & Bidochka, M. J. (2005). Serial passage of the opportunistic pathogen Aspergillus flavus through an insect host yields decreased saprobic capacity. *Canadian Journal of Microbiology*, *51*(2), 185-189. <https://doi.org/10.1139/w04-124>

Scully, L. R., & Bidochka, M. J. (2006). The host acts as a genetic bottleneck during serial infections: an insect-fungal model system. *Current Genetics*, *50*(5), 335-345. <https://doi.org/10.1007/s00294-006-0089-7>

Valero-Jimenez, C. A., van Kan, J. A. L., Koenraadt, C. J. M., Zwaan, B. J., & Schoustra, S. E. (2017). Experimental evolution to increase the efficacy of the entomopathogenic fungus Beauveria bassiana against malaria mosquitoes: Effects on mycelial growth and virulence [Article]. *Evolutionary Applications*, *10*(5), 433-443. <https://doi.org/10.1111/eva.12451>

Zhao, J., Yao, R., Wei, Y., Huang, S., Keyhani, N. O., & Huang, Z. (2016). Screening of Metarhizium anisopliae UV-induced mutants for faster growth yields a hyper-virulent isolate with greater UV and thermal tolerances. *Applied Microbiology and Biotechnology*, *100*(21), 9217-9228. <https://doi.org/10.1007/s00253-016-7746-7>

Zhou, L., Slamti, L., Nielsen-LeRoux, C., Lereclus, D., & Raymond, B. (2014). The social biology of quorum sensing in a naturalistic host pathogen system. *Current Biology*, *24*(20), 2417-2422. <https://doi.org/10.1016/j.cub.2014.08.049>
